# Revealing the pH-Dependent Adsorption Dynamics of Tetracycline Hydrochloride on Phosphoric Acid-Activated Corncob Biochar

**DOI:** 10.3390/ma19112251

**Published:** 2026-05-27

**Authors:** Qiang Zhao, Gaotian Zhao, Yalei Zhang, Yangyang Yan, Boyi Shi, Jiawei Yang, Anqi Sun, Jiabao Chen, Zongwei Zhang, Fang Wei

**Affiliations:** 1 College of Science, Civil Aviation University of China (CAUC), Tianjin 300300, China; vv10852026@163.com (Y.Z.); 13562770186@163.com (Y.Y.); shiboyi@zjnu.edu.cn (B.S.); 2 College of Aerospace Engineering, Civil Aviation University of China (CAUC), Tianjin 300300, China; 2023011103@cauc.edu.cn (G.Z.); 2023011116@cauc.edu.cn (J.Y.); anqisunan@163.com (A.S.); cjbyyyds@163.com (J.C.); 3 Science and Technology Innovation Research Institute, Civil Aviation University of China (CAUC), Tianjin 300300, China; zw-zhang@cauc.edu.cn

**Keywords:** phosphoric acid-activated biochar, adsorption isotherms, adsorption kinetic models, adsorption mechanisms, intra-particle diffusion

## Abstract

Aquaculture wastewater containing tetracycline hydrochloride (TCH) poses significant environmental problems and health risks. We investigated the adsorption of TCH onto phosphoric acid-activated corncob biochar (PCC) as a sustainable and efficient removal strategy. PCC was synthesized from cob feedstock activated by phosphoric acid under a pyrolysis temperature of 300 °C in a limited-air atmosphere. It was characterized extensively, revealing a high specific surface area (1071.75 m^2^/g), high porosity with total pore volume of 0.912 cm^3^/g, and abundant surface functional groups including phosphate, carboxylic, and amine groups. Batch adsorption experiments demonstrated an ultrahigh adsorption capacity for TCH, with a maximum theoretical capacity (Langmuir model) of 953.62 mg/g at 313 K. Its adsorption isotherms transfer from Langmuir type to Freundlich type as temperature rises, indicating a transition from monolayer to multilayer adsorption. The adsorption kinetics were governed by a synergistic mechanism involving surface adsorption and a pore-filling effect (intra-particle diffusion). Critically, the adsorption dynamics exhibit an intra-particle diffusion-controlled process at a low pH (3.0) during the final stage of adsorption. Strong hydrogen bonding led to high initial adsorption rates, and the adsorption converted to diffusion-controlled mode eventually. In contrast, at higher pH (≥7.0), electrostatic repulsion between PCC adsorbents and TCH molecules hindered intra-particle diffusion, causing the final adsorption stage to deviate from diffusion control. This work provides comprehensive insights into the pH-dependent interfacial interactions and kinetics governing TCH removal by corncob-derived, phosphoric acid-activated biochar.

## 1. Introduction

Aquaculture wastewater in fish farming contains large amounts of antibiotics like TCH, which poses a threat due to antimicrobial resistance (AMR) and environmental contamination [1]. Traditional wastewater treatments including physical adsorption by activated carbon, chemical oxidation by chlorine and ozone, and biological degradation by anaerobic treatment are usually high-cost and ineffective at removing antibiotics [2,3], leading to the development of adsorption [4], photocatalysis [5], microalgae systems [6], advanced oxidation processes [7], and their combined degradation methods [8,9,10]. Among these strategies, adsorption is a low-cost and high-efficiency method to remove antibiotics without secondary pollution [11,12,13,14]. In the past, activated carbon has been used for adsorption of antibiotics. Benefiting from abundant porous structures and an ultrahigh surface area, it has shown high adsorption capacity of pollutants in aqueous solution [15]. However, it is mainly produced from conventional fossil feedstocks with high energy consumption, high carbon emissions, and low production yield. Activated biochar is a promising alternative to activated carbon as a green adsorbent material for wastewater treatment [16,17,18]. It has several advantages like a low production cost, low energy consumption, and high efficiency in the adsorption of organic pollutants. Most importantly, it can be produced from renewable biomass carbon resources in a low-cost strategy, which is a critical pathway toward sustainable development [19,20]. Benefiting from the diversity of raw materials and flexible chemical activation strategies, the heterogeneity of biochar surface chemical groups can be achieved together with high aromaticity and a highly porous structure in adsorbent material. However, conventional alkali-activated carbon materials require an ultrahigh temperature pyrolysis process. Acidic activation also requires moderate reactivity and good thermal stability. Salts exhibit limited reactivity and poor etching ability for pore generation, which also demands restricted activation conditions. It is evident that there are some critical issues that significantly influence the adsorption process by thermal dynamic and kinetic characteristics in the research of biochar adsorbents [21]. These issues focus on: (1) morphology and surface structures of adsorbents, (2) the degree of porosity and aromaticity in the bulk structure of adsorbents, (3) surface chemical properties of the adsorbent and molecular properties of the adsorbate (especially intermolecular electro-static interaction).

In order to reveal the adsorption mechanisms of organic pollutants on activated biochar, the interactions between the adsorbent and the adsorbate must be taken into account in the analysis of adsorption’s thermodynamic and kinetic behaviors [22]. The interactions including covalent bonding, coordinate bonding, and even H-bonding with strong electron transfer are usually considered as driving forces in chemisorption, whereas interactions including non-covalent intermolecular forces such as Coulombic forces [23], π–interactions [24], dipole interactions [25], and hydrophobic interactions [26] are known to be major driving forces in physisorption. In our previous work, the adsorption process under diffusion control was proven in specific stages of adsorption of the weakly polar adsorbate molecules (with no oxygen-containing groups in its structure) on biochar [27]. In polluted water systems, there are also other polar molecules that are sensitive to pH conditions, which have several different molecular ionization forms. The diffusion process may be highly sensitive to the intermolecular force change when the solution pH value changes. However, the pH-dependent adsorption dynamics of these polar adsorbate molecules on activated biochar have not been investigated comprehensively.

In this work, we produced phosphoric acid-activated corncob biochar (PCC) at a pyrolysis temperature of 300 °C in a limited-air atmosphere. Its morphological structure, N_2_ adsorption–desorption isotherm, pore size distribution, and the surface chemistry of PCC were characterized. The adsorption isotherm models (Langmuir, Freundlich, and Temkin model) were used to simulate the adsorption data to evaluate the thermodynamic adsorption of TCH on PCC. The TCH molecular ionization forms at different pH conditions are simulated to understand the molecular charge polarity. When considering the zeta potential of PCC, the interactions of the adsorption driving forces at specific pH conditions can be reasonably inferred. Furthermore, reaction models (pseudo-first-order, and pseudo-second-order kinetic model) and intra-particle diffusion model (Weber–Morris, and Boyd model) have also been used to simulate the adsorption kinetic data by the non-linear fitting method [28]. The pH-dependent adsorption dynamics of TCH on phosphoric acid-activated corncob biochar are fully investigated with consideration of adsorption affinity (between adsorbent materials and adsorbate molecules) and intermolecular force (between adsorbate molecules) at different pH conditions.

## 2. Materials and Methods

### 2.1. Materials and Reagents

Chemical reagents, including phosphoric acid (H_3_PO_4_, AR, ≥85%), sodium hydroxide (NaOH, AR, ≥99%), and TCH were purchased from Sinopharm Chemical Reagent Co., Ltd (Beijing, China) and Tianjin Kemiou Chemical Reagent Co., Ltd (Tianjin, China). Corncob (CC) was obtained from a local market, washed thoroughly, and air-dried prior to use. Deionized water was prepared using a secondary ultrapure water system in our lab.

### 2.2. Preparation of Phosphoric Acid Activated Corncob Biochar (PCC)

PCC was synthesized by the following steps: 2.0 g of the prepared CC powder was well mixed and then uniformly impregnated with 5.0 g H_3_PO_4_ (85 wt%) for 24 h. Subsequently, it was pyrolyzed at 300 °C for 1 h in a muffle furnace at a heating rate of 5 °C min^−1^. After cooling naturally and grinding, it was stirred continuously in NaOH (0.1 mol/L) aqueous solution, filtered to obtain the solid, rinsed with deionized water to neutrality. PCC was eventually dried at 70 °C for 24 h. For comparison, water-activated corncob biochar (WCC) was also prepared by following the above method except H_3_PO_4_ was replaced by water.

### 2.3. Characterization Methods

The microscopic morphology of the products was characterized by scanning electron microscopy (SEM, JEOL-6701, JEOL Ltd., Tokyo, Japan) equipped with energy-dispersive X-ray spectroscopy (HORIBA EX-350, HORIBA Scientific, Kyoto, Japan). Pore structure parameters were determined using a JW-BK200C BET analyzer (JWGB Sci & Tech Co., Ltd., Beijing, China) via the N_2_ adsorption–desorption method at 77 K. Fourier transform infrared (FT-IR) spectra were recorded in the range of 4000–400 cm^−1^ using a Nicolet IS10 spectrometer from Thermo Fisher Scientific (Waltham, MA, USA). Simultaneous thermogravimetric (TG) and differential scanning calorimetry (DSC) analysis were performed on an SDT-Q600 instrument (TA Instruments, New Castle, DE, USA) at a heating rate of 10 °C min^−1^ from room temperature to 800 °C under air atmosphere. X-ray photoelectron spectroscopy (XPS) measurements were conducted on a Thermo ESCALAB 250xi spectrometer (Thermo Fisher Scientific, Waltham, MA, USA). The concentrations of TCH were quantified by spectrophotometry using an N5000 Plus UV-vis spectrophotometer (Youke Instruments Co., Ltd., Shanghai, China).

### 2.4. Batch Adsorption Experiments

Batch adsorption experiments were performed in centrifuge tubes by adding 10 mg of PCC into 20 mL of TCH solution with various initial concentrations (50 mg/L, 100 mg/L, 200 mg/L, 300 mg/L, 400 mg/L, 500 mg/L, 600 mg/L). The adsorption isotherms were constructed by determining the equilibrium aqueous concentration of TCH and calculating the saturated adsorption capacity of TCH (adsorbate) on PCC (adsorbent). The amount of TCH adsorbed on PCC was calculated from the mass balance Equation (1) as follows:

(1) $$q_{e}=\frac{c_{0}-c_{e}}{M}\cdot V$$

where *c*_0_ and *c*_e_ are the initial and equilibrium adsorbate concentrations (mg/L), *q*_e_ is the equilibrium adsorption capacity of adsorbate (mg/g), while *M* and *V* are the weight of the adsorbent (g) and the volume of adsorbate solution (L), respectively.

Batch adsorption kinetic experiments were performed by mixing 50 mg of PCC with 100 mL of TCH solution at a given initial concentration in a water bath thermostatic reactor. The adsorption kinetic curves (adsorption amount of TCH on PCC as a function of contact time) at various initial TCH concentrations and constant temperatures were recorded.

## 3. Results and Discussions

### 3.1. Morphology and Textural Properties of CC and PCC

**Figure 1:**
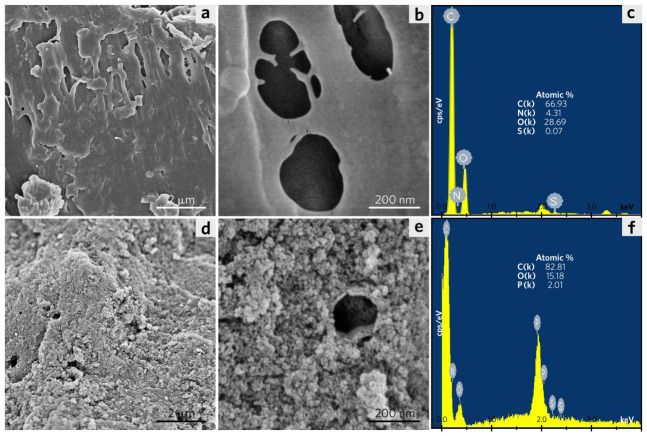
SEM images and the corresponding EDS patterns with atomic ratio of C, N, O, S, and P element of (**a**–**c**) corncob, and (**d**–**f**) PCC.

**Figure 2:**
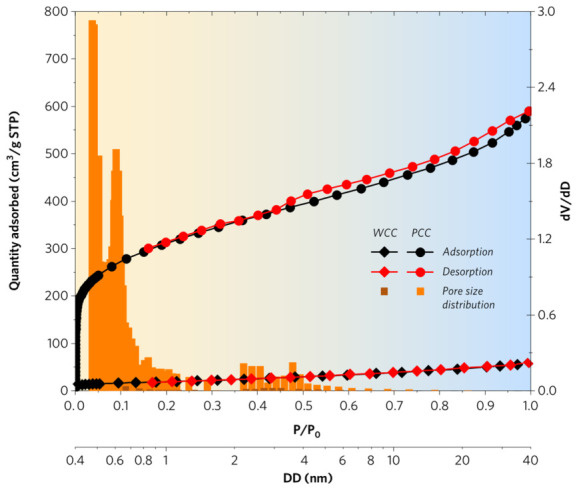
The adsorption–desorption isotherms and the pore size distribution bar charts of PCC.

The microscopic morphology and surface elemental analysis of CC and PCC were characterized by scanning electron microscopy (SEM) and energy dispersive spectrum (EDS). Figure 1a,b show a soft and smooth surface on biomass material (CC). As Figure 1c depicts, the C/O atomic ratio is 2.33 in CC. The surface becomes rough and grainy on PCC (Figure 1d,e). As a result, its C/O ratio increases to 5.45 (Figure 1f), which represents the decomposition of hydrocarbon structures from cellulose and hemicellulose at a pyrolysis temperature below 300 °C. The P element is also detected due to the formation of phosphate ester groups on the PCC surface. The BET specific surface area of CC is 1.4 m^2^/g. As shown in Figure 2 and Table 1, PCC shows a much higher specific surface area (1071.7 m^2^/g) than that of WCC (64.4 m^2^/g). Abundant micropores are formed under pyrolysis combined with H_3_PO_4_ activation, whereas WCC shows limited microporous structure as water exhibits low reactivity. Generally, porous structures are mainly generated by volatilization of H_2_O and newly formed organic volatile matter in pyrolysis. The gasification of oxidized carbon produces CO_2_, CO, CH_4_, which contributes to the formation of mesopores in PCC. High total pore volume (0.912 cm^3^/g) is supposed to be conductive to ultrahigh adsorption capacity of adsorbates on PCC.

### 3.2. Thermal Analysis of CC and PCC

**Figure 3:**
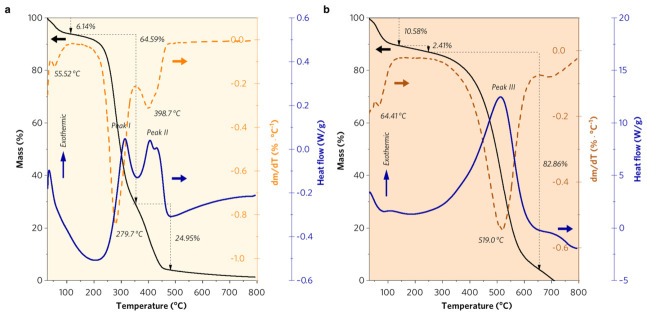
TG (black line), DTG (dashed line) and DSC (blue line) curves of CC (**a**) and PCC (**b**) in air at a heating rate of 10 °C/min within temperature range from room temperature to 800 °C. The arrow points to the coordinate axis corresponding to the curve.

The TG, DTG and DSC analysis of CC and PCC are shown in Figure 3. There are three major weight loss stages in pyrolysis of CC according to its DTG curve (Figure 3a). The first weight loss stage is attributed to the loss of adsorbed water accounting for almost 6.14% of total mass (55.52 °C). The second weight loss stage is attributed to the decomposition of hemicellulose and cellulose accounting for almost 64.59% of total mass (at 279.7 °C). The third weight loss stage is attributed to the decomposition of lignin accounting for almost 24.95% of total mass (398.7 °C). Otherwise, their corresponding differential scanning calorimetry (DSC) curve also shows several thermal reaction processes from RT to 800 °C: (1) evaporation of adsorbed water and dehydration reaction under 200 °C; (2) oxidation process of hemicellulose and cellulose at temperature range from 200 °C to 350 °C (exothermic peak I); (3) oxidation process of lignin at temperature range from 350 °C to 500 °C (exothermic peak II).

Figure 3b shows the decomposition process of PCC. The loss of adsorbed water is assigned to the DTG peak at 64.41 °C. It shows thermal stability under 300 °C, but has an obvious mass loss (its DTG peak appears at 519.0 °C) above its thermal treatment temperature in synthesis. There is a large exothermic peak III assigned to the decomposition of the carbonized composite in PCC which happens at a temperature ranging from 300 °C to 650 °C according to the DSC curves.

The phosphoric acid converts to a mixture of phosphoric acid and polyphosphoric acids, including species like H_3_PO_4_, H_4_P_2_O_7_, H_5_P_3_O_10_, or even H_n+2_P_n_O_3n+1_ (n > 4) at a temperature above 100 °C or even higher. These highly reactive polyphosphoric acids probably promote the cross-linked reaction during oxidative decomposition of biomass, inhibiting the large mass loss of organic carbon sources under the pyrolysis condition, which is dramatically different from the thermal decomposition process of CC in a finite-air atmosphere. Meanwhile, H_3_PO_4_ also exerts a mild activation effect on carbon oxidation, which generates gaseous byproducts to form abundant pores, endowing PCC with a high specific surface area.

### 3.3. Surface Chemistry of PCC

**Figure 4:**
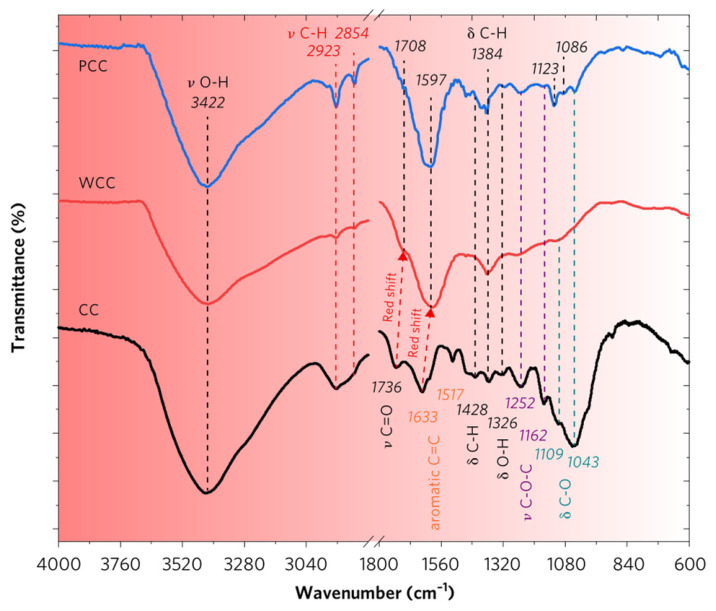
FTIR spectra of PCC, WCC and CC.

The IR spectrum of CC is shown in Figure 4, which includes absorption bands assigned to different vibration modes of several major functional groups: O–H at ~3422 cm^−1^ (ν) and 1326 cm^−1^ (δ); C–H at 2800~3000 cm^−1^ (ν) and 1350~1450 cm^−1^ (δ); ester C=O at 1736 cm^−1^ (ν); aromatic C=C at 1500~1650 cm^−1^ (δ); alcohol C–O at 1050~1100 cm^−1^ (ν); and ether C–O–C at 1150~1250 cm^−1^ (ν) [21].

The FT-IR spectra of WCC-300 and PCC are also shown in Figure 4. The absorption bands observed at 2923 cm^−1^ (ν_C–H_), 2854 cm^−1^ (ν_C–H_), 1428 cm^−1^ (δ_C–H_), and 1384 cm^−1^ (δ_C–H_) should be assigned to C–H stretching and bending vibrations of methyl (–CH_3_), methylene (–CH_2_–), and methoxy groups (–OCH_3_). The C=O absorption bands have a red shift changing from 1736 cm^−1^ (CC) to 1708 cm^−1^ (WCC and PCC), indicating the loss of ester carbonyl groups (–COOCH_3_) of pectin happens in preparation of WCC/PCC from CC [27]. It is concluded that the activation mainly accounts for the hydrolysis of the ester. The bands at 1633 cm^−1^ can be assigned to the C=C stretching vibration of the aromatic ring structure in CC (lignin) [27]. In IR spectra of WCC and PCC, this band has shifted to 1597 cm^−1^, which is due to the enhanced conjugation degree of the newly formed fused aromatic structure [27].

The broad absorption bands at 3422 cm^−1^ represent the stretching mode of O–H groups [27]. The band at 1326 cm^−1^ is evident for O–H bending vibrations of alcoholic hydroxyl groups [27]. The bands at 1252 cm^−1^ and 1162 cm^−1^ represent the C–O–C stretching vibrations of the aromatic ether of lignin and aliphatic ether of cellulose or hemicellulose, respectively [27]. By comparing raw material (CC) with the products (WCC and PCC), there are significant changes from their IR spectra. The absorption bands at 1252 cm^−1^ remain almost unchanged because the aromatic ether of lignin remains stable, whereas the absorption bands at 1162 cm^−1^ become negligible due to the decomposition of aliphatic ether of cellulose or hemicellulose.

To further reveal the activation mechanism, the spectra difference between WCC and PCC should be carefully discussed. The bands at 1109 cm^−1^ and 1043 cm^−1^ are assigned to the C–O bending mode of secondary and primary alcohol for cellulose and lignin. In the IR spectrum of WCC, these bands become smooth, which suggests the etherification and oxidation process (C–O–H transfers to C–O–C or C=O). As for PCC, the esterification process plays a dominant role in this process. The absorption bands at 1123 cm^−1^ are primarily assigned to the stretching mode of P=O, O–C stretching vibrations in P–O–C (aromatic) linkage, and P(=O)OH [29]. The shoulders at 1086 cm^−1^ are probably assigned to ionized linkage P^+^–O^−^ in phosphate esters [30]. These enhanced absorption bands also represent the generation of bonded phosphate groups in complex structures of PCC.

**Figure 5:**
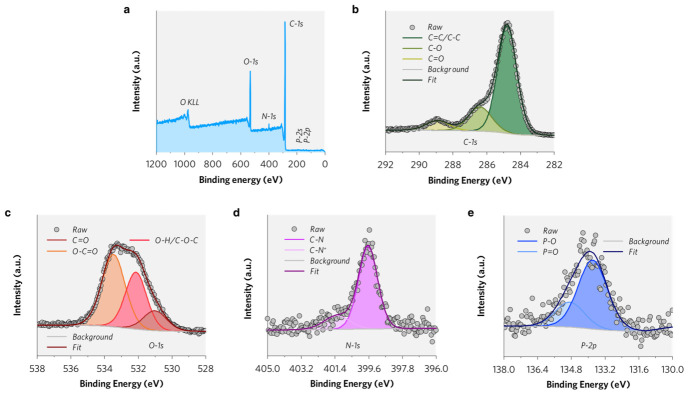
(**a**) The survey scan XPS patterns of PCC. The narrow scan XPS patterns including (**b**) C-1s, (**c**) O-1s, (**d**) N-1s, and (**e**) P-2p spectra of PCC with their deconvoluted peaks.

The X-ray photoelectron spectroscopy (XPS) analysis shows its survey scan spectrum of PCC in Figure 5a. There exist several peaks including the C-1s peak at 284.8 eV, the O-1s peak at 531.0 eV, the N-1s peak at 399.6 eV, and the P-2p peak at 134.9 eV. In Figure 5b, the narrow scan C-1s spectrum performed deconvolution of 3 overlapped peaks: peak _1 at 284.8 eV is assigned to both C–C and C=C bonds; peak_2 at 286.4 eV is assigned to C–O bonds in alcohol and ether groups; peak_3 at 288.9 eV is attributed to C=O bonds of ester and carboxylic acid [21,27]. In Figure 5c, 3 deconvolution peaks are achieved from the narrow scan O-1s spectrum. The peaks at nearly 531.0 eV (peak_1), 532.1 eV (peak_2), and 533.5 eV (peak_3) are successively assigned to O sites in C=O, O–H/C–O–C and O^*^–C=O bonds [21,27]. In Figure 5d, the narrow scan N-1s spectrum performed deconvolution of 2 overlapped peaks: peak_1 at 399.6 eV is assigned to C–N bonds; peak_2 at 401.3 eV is assigned to C–N^+^ bonds in quaternary amine groups, which are considered major chemical sites with a positive charge in acidic condition [21]. According to the deconvolution of the overlapped peaks for the narrow scan P-2p spectrum, peak_1 at 133.8 eV is ascribed as P–O and peak_2 at 134.9 eV is assigned to P=O (Figure 5e) [21,31]. The phosphate ester with terminal hydroxyl anionic groups (adsorbent–O–PO(O^−^)_2_) are considered surface chemical sites with a negative charge in weakly acidic condition.

### 3.4. Adsorption Isotherms and Thermodynamics

Three isotherm models (Langmuir, Freundlich, and Temkin equations) are used to evaluate the adsorption capacity and thermodynamics of TCH on PCC in order to understand the adsorption mechanism in depth. The experimental adsorption results are simulated by these three models via the non-linear fitting method as shown in Table 2.

The Langmuir isotherm model is put forward from a hypothesis: adsorbates are adsorbed by specific homogeneous sites within the adsorbent in the monolayer adsorption type, which can be described by the Langmuir isotherm in Equation (2):

(2) $$q_{e}=\frac{Q_{\max}\cdot K_{L}\cdot c_{e}}{1+K_{L}\cdot c_{e}}$$

where *q*_e_ is equilibrium solid phase concentration of adsorbates (mg/g), *c*_e_ is equilibrium liquid phase concentration of adsorbates (mg/L), *Q*_max_ and *K*_L_ are the Langmuir parameters related to the theoretical maximum monolayer adsorption capacity and adsorption energy, respectively.

The Freundlich isotherm model is put forward from a hypothesis: heterogeneous surface sites with a nonuniform distribution of adsorption energy within the adsorbent prevail according to the basic assumption of the Freundlich isotherm presented as Equation (3):

(3) qe=KF⋅ce1n

where *q*_e_ is equilibrium solid phase concentration of adsorbates (mg/g), *c*_e_ is equilibrium liquid phase concentration of adsorbates (mg/L), *K*_F_ and *n* are Freundlich isotherm parameters related to adsorption capacity and intensity, respectively.

**Figure 6:**
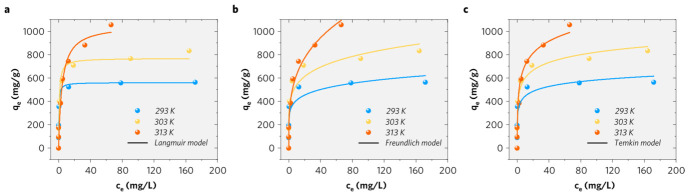
The adsorption isotherms for TCH on PCC at different temperatures (293 K in orange line, 303 K in yellow line and 313 K in blue line). Different isotherm models are used to simulate the experimental results: (**a**) Langmuir model, (**b**) Freundlich model, (**c**) Temkin model.

According to the simulated results (Figure 6a,b), the adsorption isotherms of TCH on PCC at 293 K follow the Langmuir model rather than the Freundlich model. Although the TCH adsorption isotherm at 313 K has a high adjusted R^2^ value, it exhibits a significantly increased Χr^2^ value. In general, the theoretical maximum monolayer adsorption capacity (*Q*_max_) of TCH on PCC reaches 559.9 mg/g at 293 K. It even increases to 1056.2 mg/g at 313 K. As temperature increases, the adsorption isotherm undergoes a temperature-induced transition from the Langmuir model to the Freundlich model. At low temperature (293 K), adsorbate molecules are adsorbed onto homogeneous sites of the adsorbent via monolayer adsorption. As the temperature increases, these homogeneous sites gradually transform into heterogeneous surface sites with a nonuniform distribution of adsorption energy, which means the multilayer adsorption process comes into play in this case.

The Temkin isotherm (Figure 6c) is put forward on the basis of two major assumptions: (1) uniform distribution of heterogeneous binding sites on the solid adsorbent surface and (2) linear correlation of binding energy over these different binding sites. It is expressed by Equation (4):

(4) $$\frac{q_{e}}{q_{m}}=\frac{RT}{b_{t}}\cdot \ln\left(K_{t}\cdot c_{e}\right)$$

where *q*_e_ and *q*_m_ are equilibrium and maximum solid phase concentration of adsorbates (mg/g), *c*_e_ is equilibrium liquid phase concentration of adsorbates (mg/L), *K*_t_ is the Temkin constant related to adsorption energy, *b*_t_ is related to the heat of adsorption (J/mol), *R* is the gas constant (8.314 J mol^−1^ K^−1^) and *T* is the absolute temperature (K).

Here, Equation (5) can be recast as a two-parameter expression with a *Q* term standing for *q*_m_*RT*/*b*_t_ as follows:

(5) $$q_{e}=Q\cdot \ln\left(K_{t}\cdot c_{e}\right)$$

As *q*_m_ is unable to be estimated by the Temkin model, the equilibrium adsorption capacity of PCC to TCH with an initial TCH concentration of 1000 mg/g will be used to calculate *b*_t_ (heat of adsorption) [32]. PCC exhibits a decrease in adsorption energy from 22.5 kJ/mol (293 K) to 18.5 kJ/mol (313 K). The exothermic adsorption process of TCH on PCC is hardly restricted by increasing the temperature. Due to the isotherm model change, the decrease in adsorption energy is probably attributed to the transition from monolayer to multilayer adsorption. It is speculated that the adsorption interaction forces undergo a remarkable change as temperature rises from 293 K to 313 K.

### 3.5. pH-Dependent Adsorption Affinity Between TCH and PCC

**Figure 7:**
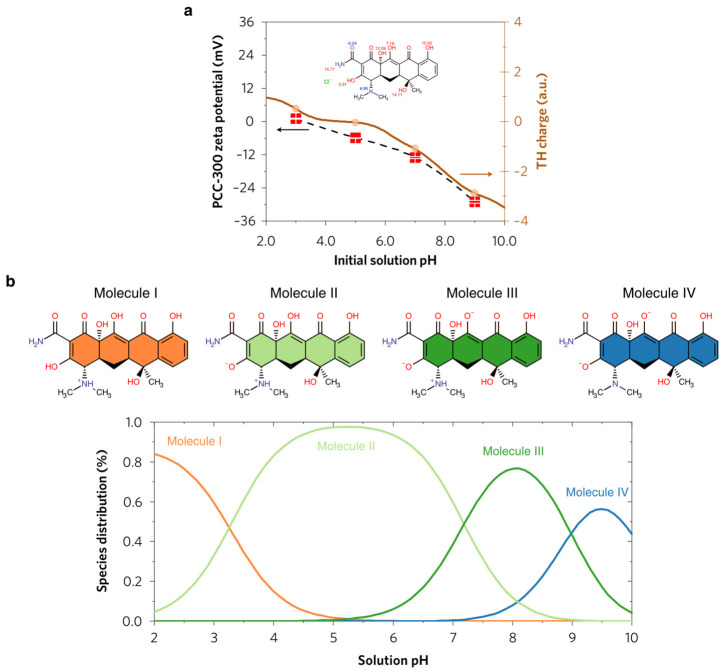
(**a**) The surface zeta potential of adsorbent and calculated molecular charge of adsorbate at different pH conditions from 3.0 to 9.0. (**b**) Molecular species percent distribution of TCH aqueous solution at different pH values.

PCC adsorbent possesses several major surface groups like phosphate groups (–C–O–P(=O)(OH)_2_, hydrogen groups (–OH), carboxylic groups (–C(=O)OH), ester groups (–C(=O)OR), and amine groups (–C–N–). As the solution pH value rises from 3.0 to 9.0, its corresponding zeta potential changes from 1.024 to −29.0 according to Figure 7a. It is accepted that the protonated amine groups contribute to their positive surface charge, and the acidic groups like phosphate and carboxylic groups lose their protons as pH rises, which contributes to their negative surface charge character at pH above 3.0.

TCH adsorbate exists in four major molecular forms as shown in Figure 7b. The TCH molecule exists as Form I and Form II in acidic condition. In general, it has a protonated quaternary ammonium at pH below ~5.0 (Form I). It converts to an inner salt molecule, which possesses a protonated quaternary ammonium and an enolate anion (Form II) together at pH between ~3.0 and ~7.0. In this stage, the hydrogen-bonding effect is considered to be the dominant intermolecular affinity.

TCH adsorbate exists as Form III and Form IV in basic condition. Enolate anions mainly contribute to the negative molecular charge as the adsorbent has a highly negative surface charge at this stage. Electrostatic repulsive force is supposed to be the intermolecular force between TCH and PCC.

### 3.6. Adsorption Kinetics

The reaction models include the pseudo-first-order (PFO) model and pseudo-second-order (PSO) model. All experimental results are simulated by the non-linear fitting method (Table S1).

#### 3.6.1. Kinetic Models of PFO and PSO

Their non-linear-form model equations are presented as Equation (6) (PFO) and Equation (7) (PSO):

(6) $$q_{t}=q_{e}-q_{e}\cdot e^{-k_{1}\cdot t}$$

(7) $$q_{t}=\frac{q_{e}^{2}k_{2}t}{1+q_{e}k_{2}t}$$

where *q*_e, *cal*_ (mg/g) and *q*_t_ (mg/g) are the amounts of adsorbates on adsorbent at equilibrium and at any contact time *t* (min); *k*_1_ (min^−1^) and *k*_2_ (g mg^−1^ min^−1^) are the adsorption rate constants of the PFO model and PSO model, respectively.

**Figure 8:**
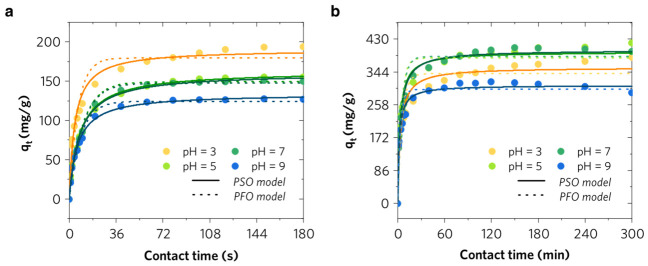
The experimental adsorption kinetic data of TCH on PCC (303 K) at different initial pH conditions (3.0–9.0) with two initial adsorbate concentrations: (**a**) 100 mg/L and (**b**) 300 mg/L.

The initial pH of TCH solution varies as its concentration changes (the initial pH is 3.85 at 100 mg/L, and it is 3.41 at 300 mg/L). In Figure 8, the initial pH of TCH solution is adjusted from 3.0 to 9.0 by adding HCl or NaOH. The additives (acid and base) dominate the solution pH throughout the entire adsorption process, from the beginning to the end (Figure S1). It changes a little until the adsorption comes to equilibrium. The adsorption kinetics follow the PSO model, but the dependence of the adsorption rate on pH differs significantly at different initial TCH concentrations.

#### 3.6.2. Elovich Models

**Figure 9:**
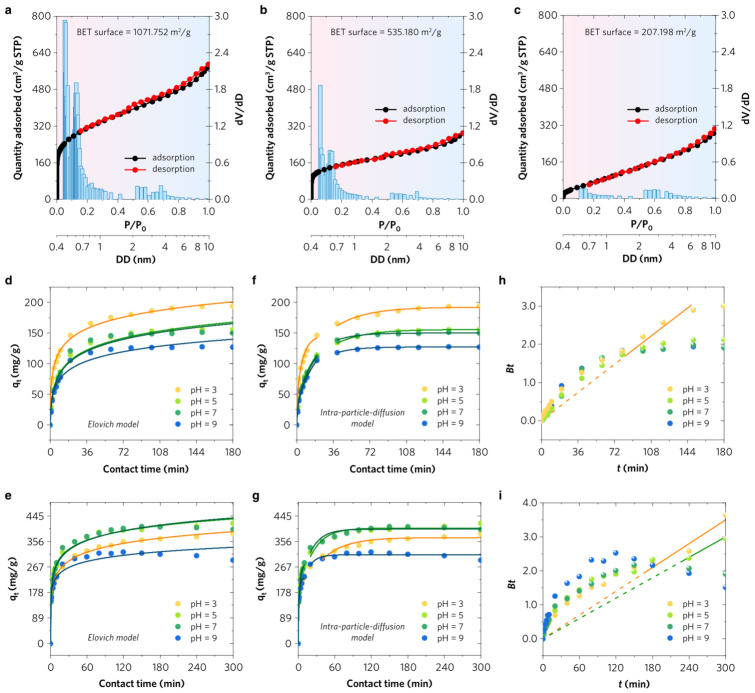
The adsorption isotherms and the pore size distribution bar charts of (**a**) PCC, (**b**) PCC after equilibrium adsorption of TCH with initial concentration of 100 mg/L, (**c**) PCC after equilibrium adsorption of TCH with initial concentration of 300 mg/L. Adsorption kinetic curves fitted by Elovich model at initial TCH adsorbate concentration of (**d**) 100 mg/L and (**e**) 300 mg/L at 313 K. Adsorption kinetic curves fitted by intra-particle diffusion model at initial TCH adsorbate concentration of (**f**) 100 mg/L and (**g**) 300 mg/L at 313 K. Boyd plots of TCH adsorption on PCC with different initial TCH concentrations of (**h**) 100 mg/L and (**i**) 300 mg/L at 313 K.

The intermolecular force and pore-filling process are supposed to be dominant factors in contribution to the adsorption dynamic. The BET surface area of PCC adsorbents with equilibrium adsorption of TCH at specific initial adsorbate concentration (100 mg/L and 300 mg/L) are measured as shown in Figure 9a–c.

The specific surface area of PCC with equilibrium adsorption of TCH at low initial TCH concentration (100 mg/L) decreases from 1071.7 m^2^/g to 535.1 m^2^/g. The mesopore volume has dramatically decreased at this stage. It is accepted that the adsorbate molecules gradually diffuse into shallow pores under concentration gradient driving force. The initial adsorption kinetic character under low concentration gradient driving force is more likely influenced by intermolecular affinity force. On the other hand, the surface area of PCC with equilibrium adsorption of TCH at high initial TCH concentration (300 mg/L) decreases from 1071.7 m^2^/g to 207.2 m^2^/g. Micropores have been almost filled by adsorbate molecules after equilibrium adsorption. To be specific, the pore filling is not negligible in the very beginning of adsorption when initial TCH adsorbate concentration is as high as 300 mg/L, which has also been proved in Section 3.6.3. As a result, the initial adsorption dynamic should be jointly controlled by intermolecular affinity force and diffusion.

In order to investigate the initial adsorption of TCH on PCC, two fundamental assumptions are accepted for the Elovich model: (1) the active energy increases with contact time and (2) heterogeneous surface properties of adsorbent. As a result, the Elovich equation can be used to estimate initial adsorption behavior and final equilibrium adsorption capacity.

The Elovich model is described by Equation (8) as follows:

(8) $$q_{t}=\frac{1}{\beta }\cdot \ln\left(1+\alpha \beta t\right)$$

where *q*_t_ (mg/g) represents the amount of adsorbate on the adsorbent at any contact time *t* (min), *α* is the initial apparent adsorption rate (mg g^−1^ min^−1^), and *β* (g mg^−1^) is related to the desorption constant.

The experimental adsorption kinetic data could be well matched by the Elovich equation with high adj. *R*^2^ values (0.997–0.952) as shown in Table S2. The apparent adsorption rate of TCH on PCC could be calculated according to the following Equation (9):

(9) $$\frac{dq_{t}}{dt}=\frac{\alpha }{1+\alpha \beta t}$$

When *t* approaches zero, the *dq*_t/_*dt* is equal to *α* (mg g^−1^ min^−1^), which represents the initial apparent adsorption rate. The intermolecular affinity between adsorbents and adsorbates mainly contributes to the initial adsorption process except for the finite pore-filling process at low initial TCH adsorbate concentration [27].

In Figure 9d, PCC exhibits the largest initial apparent adsorption rate (234.9 mg g^−1^ min^−1^) at pH = 3.0. As the pH value rises, this initial apparent adsorption rate decreases to 55.0 mg g^−1^ min^−1^ (at pH = 5.0), 53.9 mg g^−1^ min^−1^ (at pH = 7.0), and 65.1 mg g^−1^ min^−1^ (at pH = 9.0). It is evident that the oxygen-containing groups (surface–OH, surface=O, surface–COOH, surface–O–PO(OH)_2_) are presented as major surface sites of PCC. TCH molecular Form I and II also exhibit positive and neutral charge. At pH = 3.0, the hydrogen-bonding effect among these oxygen-containing groups between adsorbent and adsorbate is supposed to be strong. In Figure 9e, PCC exhibits the largest initial apparent adsorption rate (~5946.7 mg g^−1^ min^−1^) at pH = 9.0. At this stage, the pore filling significantly contributes to the initial stage of the adsorption process except for the intermolecular affinity between adsorbents and adsorbates.

#### 3.6.3. Intra-Particle Diffusion Models

Owing to the ultrahigh specific surface area of PCC, pore filling is likely to play a dominant role in the adsorption kinetic process of TCH onto PCC. Especially at high initial TCH concentrations, an extremely high initial apparent adsorption rate was observed based on Elovich model analysis. To be specific, the adsorption of matter from aqueous solutions onto porous adsorbents generally involves three sequential steps: (1) diffusion of the adsorbate molecules through the boundary layer around the adsorbent particle (external film diffusion); (2) diffusion of adsorbate molecules through the pore structure which may be due to pore diffusion or surface diffusion or a combination of both (intra-particle diffusion); (3) adsorption on the internal pore surface (surface reaction). In most cases, the surface reaction step is faster than the diffusion step [33]. In a well-designed real-time sampling reactor with forced stirring, the dye concentration gradient in the liquid film is negligible, which can largely reduce or even eliminate the film mass transfer resistance, so film diffusion can be negligible in these adsorption systems in our work [27]. In order to understand how the intra-particle mass transfer (intra-particle diffusion) influences the adsorption kinetic process, a diffusion model based on Fick’s law is applied with an assumption of a spherical adsorbent particle with an average radius of *R*. The relationship between the dye adsorption quantity (*q*_t_) and contact time (*t*) is given by Equation (10):

(10) $$\frac{q_{t}}{q_{\infty }}=1-\frac{6}{\pi^{2}}\sum_{n=1}^{\infty }\frac{1}{n^{2}}\exp\left(-\frac{D_{i}n^{2}\pi^{2}t}{R^{2}}\right)$$

where *q*_∞_ (mg/g) represents the equilibrium adsorption quantity in the solid phase of adsorbent at infinite time, *R* is the average radius of the spherical adsorbent particle, *D_i_* (cm^2^/s) is the diffusion coefficient.

If *B* is equal to *D_i_* π^2^/R^2^, Equation (11) can be simplified as below:

(11) $$\frac{q_{t}}{q_{\infty }}=1-\frac{6}{\pi^{2}}\sum_{n=1}^{\infty }\frac{1}{n^{2}}\exp\left(-n^{2}Bt\right)$$

For a short time, when *q*_t_/*q*_∞_ < 0.3, Equation (11) can be simplified to yield:

(12) $$\frac{q_{t}}{q_{\infty }}=\frac{6}{\pi^{1.5}}\cdot {\left(Bt\right)}^{0.5}$$

which is usually called the Weber–Morris model plotting *q*_t_ versus *t*^0.5^. If it shows a straight line passing through the origin, it means that the adsorption process is controlled by intra-particle diffusion.

For a moderate time, when *q*_t/_*q*_∞_ < 0.85, Equation (11) can be simplified to yield:

(13) $$\frac{q_{t}}{q_{\infty }}=\frac{6}{\pi^{1.5}}\cdot {\left(Bt\right)}^{0.5}-\frac{3}{\pi^{2}}Bt$$

For a long time, when *q*_t/_*q*_∞_ > 0.85, Equation (11) can be simplified to yield:

(14) $$\frac{q_{t}}{q_{\infty }}=1-\frac{6}{\pi^{2}}\exp\left(-Bt\right)$$

Equations (13) and (14) are known as the Boyd or Reichenberg model.

According to Figure 9f,g, the experimental adsorption kinetic data can be theoretically simulated by those above piece-wise intra-particle diffusion Equations (13) and (14) (Table S3). These adsorption kinetic curves are well matched with the intra-particle diffusion model in the initial stage of the adsorption process. As B is responsible for the diffusion coefficient (*D_i_*), it is estimated to be 0.2565–0.3645 min^−1^ at high initial TCH concentration (300 mg/L), which is much larger than that at low initial TCH concentration (100 mg/L). These results are consistent with the hypothesis and simulated results in Elovich model analysis.

To investigate how the intra-particle diffusion contributes to the kinetic adsorption process at different pH, the plots of *Bt* vs. *t* are shown in Figure 9h,i, which are called the Boyd plot. It means that the adsorption process is controlled by intra-particle diffusion if it shows a straight line passing through the origin point. In the initial adsorption stage (within 10 min), the intra-particle diffusion is supposed to be a dominant process governing the adsorption kinetic character. The process gradually transitions to non-diffusion-controlled behavior with increasing contact time. Only at pH below 3.0 is the final adsorption stage probably dominated by the intra-particle diffusion. At high initial TCH concentration, a high concentration gradient tends to govern the diffusion-controlled adsorption at the final stage when initial pH increases to 5.0.

The TCH adsorbate molecules are mainly Form I and Form II at pH = 3.0, the intermolecular interaction is weak repulsive force. The main stage of adsorption, except for the initial stage, deviated from the diffusion control at pH above 7.0 due to the strong repulsive intermolecular force. It suggests the strong repulsive intermolecular force is not favorable for diffusion-controlled adsorption except for the adsorption at the very beginning.

## 4. Conclusions

In summary, we investigated the pH-dependent adsorption dynamics of TCH on phosphoric acid-activated corncob biochar with consideration of adsorption affinity (between adsorbent materials and adsorbate molecules) and intermolecular forces (between adsorbate molecules) at different pH conditions.

The adsorption of TCH on PCC exhibits a physisorption-like process due to the significant decrease in the simulated heat of adsorption from the Temkin model. Its adsorption isotherms transfer from Langmuir-type to Freundlich-type as temperature rises. This is probably attributed to the enhanced adsorbate molecular aggregation induced by attractive Coulombic force and the dipole–dipole H-bonding effect.

The adsorption kinetic behavior of TCH on PCC exhibits a synergetic mechanism of surface adsorption (active sites) and pore-filling (intra-particle diffusion). At the very beginning stage of adsorption, it is triggered by adsorption affinity on the external surface of the adsorbent. At the final stage, the pore-filling is the primary driving force, which is attributed to the intra-particle diffusion in the rate-controlling step.

As solution pH rises from 3.0 to 9.0, the enhanced desorption and hindrance effect by the repulsive Coulombic force between adsorbent materials and adsorbate molecules become key factors causing the adsorption kinetics to be out of diffusion control at the final stage of adsorption.

## Figures and Tables

**Table 1:** Textural and pore structural parameters of PCC.

Sample Name	*S*_BET_ m^2^/g	*S*_mesopore_ m^2^/g	*V*_total pore_ cm^3^/g	*V*_micropore_ cm^3^/g	*V*_mesopore_ cm^3^/g	Mid-Value *D*_micropore_ nm	Average *D*_total_ nm
**CC**	1.4	–	–	–	–	–	–
**W** **CC**	64.4	72.6	0.092	0.027	0.096	0.508	5.740
**PCC**	1071.7	637.5	0.912	0.430	0.710	0.634	3.405

Note: CC has negligible surface area with no specific pore structure. *S***_BET_**: BET specific surface area; V: pore volume; D: pore diameter.

**Table 2:** Adsorption isotherm models in simulation for adsorption of TCH on PCC.

Isotherm Model	Temperature	Parameters	PCC	Temperature	Parameters	PCC
Langmuir	293 K	*Q*_max_	559.89	303 K	*Q*_max_	768.30
		*K*_L_	2.601		*K*_L_	1.214
		Χ_r_^2^	1832.9		Χ_r_^2^	2859.7
		Adj. R^2^	0.955		Adj. R^2^	0.973
	313 K	*Q*_max_	1056.22			
		*K*_L_	0.230			
		Χ_r_^2^	4793.9			
		Adj. R^2^	0.968			
Freundlich	293 K	*K*_F_	292.07	303 K	*K*_F_	358.40
		*n*	6.856		*n*	5.604
		Χ_r_^2^	10,227.9		Χ_r_^2^	9423.6
		Adj. R^2^	0.752		Adj. R^2^	0.911
	313 K	*K*_F_	319.74			
		*n*	3.407			
		Χ_r_^2^	3506.6			
		Adj. R^2^	0.977			
Temkin	293 K	*Q*	60.53	303 K	*Q*	96.65
		*K*_t_	149.34		*K*_t_	48.56
		*b*_t_ (kJ/mol)	22.5		*b*_t_ (kJ/mol)	20.0
		Χ_r_^2^	6709.9		Χ_r_^2^	2505.4
		Adj. R^2^	0.837		Adj. R^2^	0.976
	313 K	*Q*	148.80			
		*K_t_*	11.65			
		*b*_t_ (kJ/mol)	18.5			
		Χ_r_^2^	3729.3			
		Adj. R^2^	0.975			

Note: All these parameters are simulated via non-linear fitting method.

## Data Availability

The original contributions presented in this study are included in the article/Supplementary Material. Further inquiries can be directed to the corresponding authors.

## Supplementary Materials

The following supporting information can be downloaded at: https://www.mdpi.com/article/10.3390/ma19112251/s1, **Figure S1**: The pristine solution pH values of different initial TCH concentrations and the pH values when it comes to equilibrium adsorption on PCC at room temperature. **Table S1**: The estimated kinetic parameters of PFO and PSO model by non-linear method in adsorption of TCH on PCC at different pH conditions at 293 K. **Table S2**: The estimated kinetic parameters of Elovich model by non-linear method in adsorption of TCH on PCC at different pH conditions at 293 K. **Table S3**: The estimated kinetic parameters of intra-particle diffusion model by non-linear method in adsorption of TCH on PCC at different pH conditions at 293 K. **Table S4**: The specific surface area, TCH adsorption capacity, activation method between PCC and other reported biochars [4,12,13,34,35,36,37].
