# Supplementary material for: Revealing the pH-Dependent Adsorption Dynamics of Tetracycline Hydrochloride on Phosphoric Acid-Activated Corncob Biochar
Source: Materials (Basel). 2026 May 27;19(11):2251. doi: 10.3390/ma19112251 (PMC13258645; doi:10.3390/ma19112251)
Supplement: Supplementary file 1 [file materials-19-02251-s001.zip › materials-4268001-supplementary.pdf]

Supporting Information

Revealing the pH-dependent adsorption dynamics of tetracycline hydrochloride on phosphoric acid-activated corncob biochar

Qiang Zhao[1, 2, *], Gaotian Zhao[2], Yalei Zhang[1], Yangyang Yan[1], Boyi Shi[1], Jiawei Yang[2], Anqi Sun[2], Jiabao Chen[2], Zongwei Zhang[3], Fang Wei[1, 2, *]

[1] College of Science, Civil Aviation University of China (CAUC), Tianjin, 300300, China

[2] College of Aerospace Engineering, Civil Aviation University of China (CAUC), Tianjin, 300300, China

[3] Science and Technology Innovation Research Institute, Civil Aviation University of China (CAUC), Tianjin, 300300, China

* Corresponding author. College of Science, Civil Aviation University of China (CAUC), Tianjin, 300300, China

* Email address: zhao-q@cauc.edu.cn (Q. Zhao). f_wei@cauc.edu.cn (F. Wei).

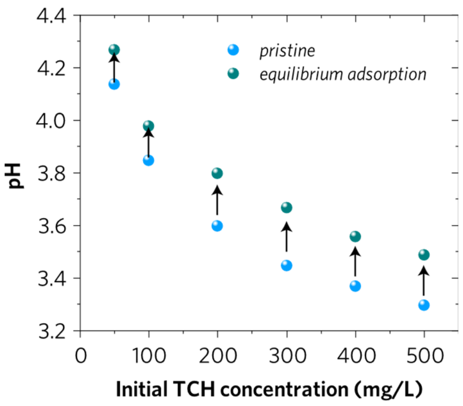

Figure S1 The pristine solution pH values of different initial TCH concentrations and the pH values when it comes to equilibrium adsorption on PCC at room temperature.

Table S1 The estimated kinetic parameters of PFO and PSO model by non-linear method in adsorption of TCH on PCC at different pH conditions at 293 K.

Kinetic model	***parameters***	***c_0 = 100 mg/L***				***c_0 = 300 mg/L***			
		pH = 3	***pH = 5***	***pH = 7***	***pH = 9***	***pH = 3***	***pH = 5***	***pH = 7***	***pH = 9***
Pseudo-first-order	$q_{e,\ cal}$	*179.53*	*149.27*	*147.42*	*124.44*	*339.98*	*380.74*	*384.33*	*298.52*
	k_1	*0.151*	*0.096*	*0.097*	*0.115*	*0.180*	*0.209*	*0.187*	*0.274*
	Adj. R^2	*0.890*	*0.969*	*0.984*	*0.981*	*0.841*	*0.846*	*0.910*	*0.870*
Pseudo-second-order	$q_{e,\ cal}$	*190.40*	*163.06*	*160.64*	*134.11*	*356.08*	*396.64*	*401.30*	*309.14*
	k_2	*0.0012*	*0.0008*	*0.0008*	*0.0012*	*0.0008*	*0.0009*	*0.0008*	*0.0016*
	Adj. R^2	*0.954*	*0.992*	*0.992*	*0.994*	*0.931*	*0.940*	*0.972*	*0.954*

Table S2 The estimated kinetic parameters of Elovich model by non-linear method in adsorption of TCH on PCC at different pH conditions at 293 K.

Kinetic model	***parameters***	***c_0 = 100 mg/L***				***c_0 = 300 mg/L***			
		pH = 3	***pH = 5***	***pH = 7***	***pH = 9***	***pH = 3***	***pH = 5***	***pH = 7***	***pH = 9***
Elovich	α	*234.93*	*55.00*	*53.89*	*65.12*	*975.34*	*1919.95*	*1288.07*	*5946.71*
	β	*0.036*	*0.035*	*0.035*	*0.045*	*0.023*	*0.022*	*0.020*	*0.033*
	Adj. R^2	*0.994*	*0.986*	*0.968*	*0.968*	*0.997*	*0.993*	*0.978*	*0.952*

Table S3 The estimated kinetic parameters of intra-particle-diffusion model by non-linear method in adsorption of TCH on PCC at different pH conditions at 293 K.

Kinetic model	*parameters*	*c_0 = 100 mg/L*				*c_0 = 300 mg/L*			
		pH = 3	*pH = 5*	*pH = 7*	*pH = 9*	*pH = 3*	*pH = 5*	*pH = 7*	*pH = 9*
**Boyd** $q_t/q_\infty < 0.85$	q_∞	148.86	207.13	231.86	222.39	239.35	277.78	288.97	238.52
	B	0.1372	0.0194	0.0138	0.0133	0.3538	0.3912	0.2565	0.3645
	Adj. R^2	0.967	0.997	0.994	0.993	0.976	0.985	0.994	0.972
**Boyd** $q_t/q_\infty > 0.85$	q_∞	192.33	155.79	149.97	127.13	369.13	402.24	398.72	309.36
	B	0.0338	0.0361	0.0519	0.0534	0.0272	0.0448	0.0547	0.0837
	Adj. R^2	0.924	0.994	0.964	0.984	0.850	0.859	0.768	0.553

Table S4. The specific surface area, TCH adsorption capacity, activation method between PCC and other reported biochars.

biomass	*Activation method of biochar*	S_{BET} (m²/g)	*Adsorption temperature(°C)*	*Langmuir model* q_{max} (mg/g)	*Ref.*
Corn cob	*H_3PO_4-activation and pyrolysis at 300 °C*	1071.7	20	559.89	*This article*
Corn cob	*H_3PO_4-activation and pyrolysis at 300 °C*	1071.7	30	768.26	*This article*
Corn cob	*H_3PO_4-activation and pyrolysis at 300 °C*	1071.7	40	1056.22	*This article*
Chlorella vulgaris	*P-doped KOH activattion at 700°C under N_2*	1873.78	30	662.91	[12]
Wheat stalk	*Pyrolysis at 600 °C under N_2 and ball milled*	257.70	25	75.95	[4]
Barley straw	*$ZnCl_2$-activation and pyrolysis at 750 °C under N_2*	862.85	25	531.41	[34]
Barley straw	*H_3PO_4-activation and pyrolysis at 750 °C under N_2*	404.58	25	252.37	[34]
Willow catkin	*Pyrolysis at 600 °C then mixing with KOH pyrolysis at 800 °C under N_2*	2402.31	45	1242.31	[35]
Enteromorpha prolifera	*Copyrolysis at 900 °C under N_2 with cobalt nitrate*	-	25	940.44	[13]
Sugarcane bagasse	*Mixed with $NaHCO_3$, melamine and red mud pyrolyzed at 700°C under N_2*	279.1	30	143.9	[36]
Banana peel	*ZIF-8 functionalized biochar pyrolysis at 700°C under N_2*	1200	20	288.85	[37]

References:

[4] Xiang W, Wan Y, Zhang X, et al. Adsorption of tetracycline hydrochloride onto ball-milled biochar: Governing factors and mechanisms[J]. Chemosphere, 2020, 255: 127057.

[12] Liu B, Li W, Huang Y, et al. Enhanced tetracycline hydrochloride removal by ultra-microporous phosphorus-doped KOH-activated microalgal biochar: Adsorption performance and mechanistic insights[J]. Environmental Research, 2025: 123040.

[13] Xiao D, Han R, Ai L, et al. Efficient removal of tetracycline hydrochloride by cobalt-modified Enteromorpha prolifera derived biochar: Synergy of adsorption and sulfite activation[J]. Separation and Purification Technology, 2025: 134435.

[34]Song J, Xi H, Gu X, et al. Study on the Adsorption of Tetracycline Hydrochloride in Water by Modified Highland Barley Straw Biochar[J]. Water, 2025, 17(23): 3335.

[35] Wei T, Song X, Zhang J, et al. Efficient adsorption of tetracycline hydrochloride by Willow Catkins based biochar: performance, governing factors and mechanisms[J]. Biomass Conversion and Biorefinery, 2024, 14(16): 18761-18773.

[36] Zhang L, Xiao J, Che H, et al. Novel magnetic N-doped biochar derived from sugarcane bagasse and red mud for effective adsorption of tetracycline hydrochloride[J]. Journal of Environmental Chemical Engineering, 2024, 12(3): 113041.

[37] Liu J, Zeng Q, Chen Y, et al. Synthesizing ZIF-8 functionalized biochar by in situ reuse of residual Zn from chemical activation for enhanced tetracycline hydrochloride adsorption removal[J]. Journal of Environmental Chemical Engineering, 2025, 13(3): 116729.
